# Predictors of male condom use among sexually active heterosexual young women in South Africa, 2012

**DOI:** 10.1186/s12889-018-6039-8

**Published:** 2018-09-24

**Authors:** Thobani Ntshiqa, Alfred Musekiwa, Mandla Mlotshwa, Kerry Mangold, Carl Reddy, Seymour Williams

**Affiliations:** 1 South African Field Epidemiology Training Programme (SAFETP), National Institute For Communicable Disease (NICD), Sandringham, Johannesburg, South Africa; 2 South African National Aids Council (SANAC), Pretoria, South Africa; 3 School Of Health Systems And Public Health, University Of Pretoria, Pretoria, South Africa; 4 Centers for Disease Control and Prevention, Pretoria, South Africa; 5 Division of Global Health Protection, Center for Global Health, Centers for Disease Control and Prevention, Atlanta, USA

**Keywords:** Predictor, Condom use, Young women, Sexually active, HIV prevention, South Africa

## Abstract

**Background:**

In South Africa, young women are at disproportionate risk of HIV infection with about 2363 new infections per week in 2015. Proper condom use is one of the most effective HIV/AIDS prevention strategies among sexually active persons. Understanding factors associated with male condom use in this key population group is important to curb the spread of HIV. This study determined practices and predictors of male condom use among sexually active young women in South Africa.

**Methods:**

The 2012 National HIV Communication Survey measured the extent of exposure to communication activities for HIV prevention among men and women aged 16–55 years in South Africa. We performed a secondary data analysis on a subset of this survey, focussing on 1031 women aged 16–24 years who reported having had sex in the past 12 months. We determined predictors of male condom use using the unconditional multivariable logistic regression model.

**Results:**

Of the 1031 young women, 595 (57.8%) reported using a male condom at last sex, 68.4% in women aged 16–19 years and 54.5% in women aged 20–24 years (*p* < 0.001). Delayed sexual debut [20 years or above] (Adjusted Odds Ratio [aOR] 2.1, 95% CI: 1.2 to 3.7, *p* = 0.006); being a student (aOR 1.6, 95% CI: 1.2 to 2.3, *p* = 0.005); and exposure to HIV communication programmes (aOR 3.1, 95% CI: 1.2 to 8.6, *p* = 0.025) were significantly associated with male condom use at last sex.

**Conclusion:**

Male condom use was a common practice among young women and was associated with delayed sexual debut and exposure to HIV communication programmes. Behavioral interventions and HIV communication programmes should therefore encourage young women to delay initiation of sex and promote usage of male condoms.

**Electronic supplementary material:**

The online version of this article (10.1186/s12889-018-6039-8) contains supplementary material, which is available to authorized users.

## Background

Sub-Saharan Africa is the epicenter of the HIV/AIDS epidemic, with more than 70% of all people living with HIV residing in this region [1]. Although the number of new HIV infections is declining steadily in sub-Saharan Africa and in other parts of the world, South Africa continues to have the highest incidence of HIV in the world with sexually active women being the most affected group [2, 3]. Compared to their male counterparts, young women bear a disproportionate share of the HIV epidemic in South Africa with a prevalence of 5.6% among those aged 15–19 years and 17.4% among those aged 20–24 years [4]. Furthermore, young women acquire HIV infection five to seven years earlier than men and are three to six times more likely to become infected compared with young men in the same age group [4]. Heterosexual HIV transmission accounts for more than 90% of new HIV infections among sexually active people with most infections attributed to not using a male condom [1, 5].

Male condom use is one of the most effective HIV prevention strategies among sexually active people [1, 3]. Countries that report a higher use of condoms in population based surveys have consistently reported lower HIV prevalence as compared to those countries that report lower use of condoms [3]. Numerous studies in sub-Saharan Africa have provided compelling scientific evidence to support this notion [2, 3, 6]. Condom use has been shown to be effective in preventing HIV and sexually transmitted infection (STIs) by more than 90% [3, 6, 7]. Data from the South African household survey on HIV prevalence, incidence and behaviour report indicates that increasing number of adolescents initiate sexual activities early, with multiple partners, and inconsistently use condoms [4]. Increase in male condom use in South Africa played a critical role in the decline in HIV incidence that occurred between 2000 and 2008, highlighting a continued need to invest in male condom promotion and distribution [2]. However, data among adolescents and young women in South Africa indicates that condom use is under-utilized in this key population, who remain highly vulnerable to HIV [8]. Consequently, to date South Africa has one of the highest HIV prevalences in the world with 12.2% (6.4 million persons) of the population being HIV-infected in 2012 [2–4]. The burden of HIV varies considerably across various demographic and geographic areas within the nine provinces of South Africa [4]. KwaZulu-Natal has the highest HIV prevalence (17.4%) followed by Free State (14.7%), Mpumalanga (14.5%), North West (13.9%), Gauteng (12.8%), Eastern Cape (12.2%), Limpopo (9.4%), Northern Cape (7.8%), and the Western Cape having the lowest HIV prevalence (5.1%) [4]. Compared to their male counterparts, women bear a higher burden of the HIV epidemic in South Africa (14.4% vs. 9.9%, *p* < 0.001) which starts to emerge among 15–19 year olds [4].

While new HIV infections among sexually active young women are largely attributed to not using a male condom during sexual intercourse, predictors of male condom use are not well understood [2, 4, 9]. Identifying predictors of male partner condom use in sexually active young women is crucial to inform HIV prevention programmes to mitigate HIV risk factors. Lack of female power in sexual relationships, threat of physical violence if condom use is requested, negative stigma associated with condoms and financial incentives to forego condom use are amongst factors identified as potential barriers to condom use [10]. Condom use have also been associated with higher levels of education, belief that condoms did not reduce sexual pleasure, believing that condoms were safe and having multiple sexual partners [11]. In addition, being married or being in a cohabiting relationship, and equating condom use with lack of trust have been shown to decrease the likelihood of condom use at last sex [11]. Data from Brazil and Cambodia studies also found that condom use was associated with type of sexual partner, whether casual or regular, and household’s socio-economic status of young women [12, 13]. In KwaZulu-Natal, South Africa, Chimbindi et al. found that condom use was associated with living separately from a sexual partner and belonging to a household with a higher socio-economic status [9]. They also found that young women who have regular partners, are older age and those in relationship with older partners were less likely to use condom at last sexual encounter [9]. In South Africa and many Sub-Saharan African countries, delayed sexual debut, casual sex and multiple sexual partnership were found to be major predictors of male condom use at last sex [9, 14–17].

In combination with other HIV prevention strategies, male condom use is widely reported to lower the HIV transmission risk. Understanding factors associated with male condom use is important to inform targeted interventions and mitigate risks for HIV infection in this population group. In this study we described the male condom use practices and determined demographic factors, sexual behavioural practices, and exposure to behavioural change communications associated with male condom use among sexually active women aged 16–24 years in South Africa. We also determined demographic and sexual behavioural practices predicting HIV infection among sexually active women aged 16–24 years in South Africa.

## Methods

### Study design

This study involved secondary data analysis of the 2012 National HIV Communication Survey database from a cross-sectional study design [18]. The data set used in this analysis is a subset of the bigger survey which included men and women aged 16–55 years. This study only included women aged between 16 and 24 years in order to achieve the specific objectives for this study.

### Study site and sampling methods

The 2012 National HIV Communication Survey included a proportionally representative sample of men and women from all the provinces, districts, and sub-districts of South Africa across all population groups. A multi-stage cluster sampling approach with three stages was used. Firstly, the sample size calculation ensured proportional representation of the population size in each province. Within each province, the primary sampling unit was selected on probability proportional to the population in each sub-district. From each province the first sub-district was randomly selected, thereafter additional sub-districts were selected from each province by systematically skipping through the listed sub-districts in each province according to a sampling interval that yielded the desired sample. Secondly, households in each sub-district were identified for a visit. For each sub-district, a sampling interval was calculated and the starting point was randomly selected. Finally, one eligible respondent per household was randomly selected to be interviewed [18].

### Study population

The study participants were women aged 16–24 years who reported having had sex in the past 12 months. All races were represented and participants were categorized into two age groups (16–19 years and 20–24 years).

### Inclusion criteria

The eligible participants for inclusion in this analysis were South African women and girls between the ages of 16 to 24 years who reported ever had sex and had sex in the past 12 months at enrolment.

### Sample size

The 2012 National HIV Communication Survey recruited 5969 women aged 16–55 years. To ensure proportional representation of the South African population with respect to urban and rural dwelling, sex, population group, age, and province, the final sample was weighted using the 2007 Community Survey conducted by Statistics South Africa [18]. The total number of women aged 16–24 years was 1922. Among these, 1888 (98.2%) of the women reported ever having had sex. This study included 1031 (54.6%) women who reported having had sex in the previous 12 months.

### Data collection tools

A structured questionnaire was administered by a fieldworker to each participant focusing on demographics, knowledge, attitudes, perceptions, sexual behaviour and exposure to HIV communication programmes in the 2012 National HIV Communication Survey [18]. We utilised secondary data obtained from this survey and our analysis focused on demographic characteristics, sexual behavioural practices, self-reported HIV counseling and testing practices and exposure to HIV communication programmes.

### Data analysis

A nationally representative data set from the 2012 National HIV Communication Survey was analyzed using Stata version 13 (StataCorp. 2013. Stata Statistical Software: Release 13. College Station, TX: StataCorp LP). The socioeconomic status was created on Stata and measured using the principal components analysis (PCA). We further used cluster analysis on the household socio-economic scores to categorize the distribution into ‘low’, ‘medium’ and ‘high’ socio-economic groups applying arbitrary cut-off points (40–40–20 split) [19]. Socio-demographics and sexual behaviour factors were direct questions from the survey and were described using frequencies (n) and percentages (%), and Chi-square (χ^2^) test or two-sided Fisher’s exact test were used to compare categorical variables. For statistical comparisons, a *p* ≤ 0.05 was considered significant. The bivariate and multivariable logistic regression models were used to determine predictors or factors independently associated with male condom use or HIV infection. We adjusted all the logistic regression models using the **svy** prefix command in Stata, which adjusts for survey weights using survey design variables. Manual forward stepwise procedure was used to select variables for the multivariable model. Predictors with *p* < 0.15 in the bivariate logistic regression models were introduced into the multivariable model. A cut-off *p*-value of less than 0.05 was used to retain variables in the final multivariable model. Results were summarized using Odds Ratios (OR) and Adjusted Odds Ratios (aOR) with their corresponding 95% Confidence Intervals (CI) and *p*-values.

### Ethical clearance & permission

The 2012 National HIV Communication Survey was approved by the University of Witwatersrand Human Research Ethics Committee (Non-Medical) (H110701) and the Institutional Review Board of the Johns Hopkins Bloomberg School of Public Health [17]. Ethical approval for the secondary data analysis presented in this study was granted by the Center for Global Health at the Center for Disease Control and Prevention and University of Pretoria Research Ethics Committee (180/2015) and no informed consent was required as this study is a secondary data analysis. The Deputy Director of Monitoring and Evaluation Research at Johns Hopkins Health and Education in South Africa (JHHESA) granted the permission to conduct this study and access the 2012 National HIV Communication Survey data.

## Results

### Sociodemographic characteristics

This study included 1031 women who reported having had sex in the past 12 months with a median age of 21 years (IQR: 20; 23). Of this, 24.0% (247/1031) were 16–19 years old and 76.0% (784/1031) were aged 20–24 years (Table 1). The majority of the respondents were Black African women (87.8%, 905/1031), either single (41.7%, 430/1031) or in stable relationships (37.0%, 381/1031). More than two-third [71.0% (urban formal (33.1%, 341/1031); urban informal (37.9%, 391/1031)] reported living in an urban setting, and to have attended grade 11 (44.2%, 455/1031) and matric (43.2%, 445/1031). Almost 60 % (58.8%, 606/1031) reported that they were unemployed with 42.8% (441/1031) reportedly from households with medium socio-economic status (Table 1).

**Table 1:** Demographic characteristics of sexually active young women aged 16–24 years, National HIV Communication Survey, South Africa, 2012

Variable	Age groups		
Age	16–19 years - *n* (%)	20–24 years - *n* (%)	N (%)
	247 (24.0%)	784 (76.0%)	1031 (100.0%)
Race			
Black	213 (86.2%)	692 (88.3%)	905 (87.8%)
Coloured	33 (13.4%)	81 (10.3%)	114 (11.1%)
White	1 (0.4%)	9 (1.2%)	10 (1.0%)
Indian	0 (0.0%)	2 (0.3%)	2 (0.2%)
Marital status			
Single	127 (51.4%)	303 (38.7%)	430 (41.7%)
Stable relationship	92 (37.3%)	289 (36.9%)	381 (37.0%)
Cohabitation	20 (8.1%)	12 (15.3%)	140 (13.6%)
Married, staying with husband	1 (0.4%)	49 (6.3%)	50 (4.9%)
Married, not staying with husband	1 (0.4%)	10 (1.3%)	11 (1.1%)
Divorced	0 (0.0%)	2 (0.3%)	2 (0.2%)
Other	6 (2.4%)	11 (1.4%)	17 (1.7%)
Province			
Eastern Cape	19 (7.7%)	73 (9.3%)	92 (8.9%)
Free State	22 (8.9%)	67 (8.6%)	89 (8.6%)
Gauteng	49 (19.8%)	150 (19.1%)	199 (19.3%)
KwaZulu-Natal	43 (17.4%)	161 (20.5%)	204 (19.8%)
Limpopo	19 (7.7%)	81 (10.3%)	100 (9.7%)
Mpumalanga	33 (13.4%)	81 (10.3%)	114 (11.1%)
North West	18 (7.3%)	47 (6.0%)	65 (6.3%)
Northern Cape	8 (3.2%)	13 (1.7%)	21 (2.0%)
Western Cape	36 (14.6%)	111 (14.2%)	147 (14.3%)
Settlement type			
Urban formal	84 (34.0%)	257 (32.8%)	341 (33.1%)
Urban informal	92 (37.3%)	299 (38.1%)	391 (37.9%)
Peri-urban	29 (11.7%)	96 (12.2%)	125 (12.1%)
Tribal settlement	35 (14.2%)	116 (14.8%)	151 (14.7%)
Farming	7 (2.8%)	16 (2.0%)	23 (2.2%)
Education			
No schooling	0 (0.0%)	2 (0.3%)	2 (0.2%)
Primary	9 (3.7%)	27 (3.4%)	36 (3.5%)
Grade 11	148 (60.2%)	307 (39.2%)	455 (44.2%)
Matric	80 (32.5%)	365 (46.6%)	445 (43.2%)
Tertiary	9 (3.7%)	83 (10.6%)	92 (8.9%)
Employment status			
Unemployed	94 (38.1%)	512 (65.3%)	606 (58.8%)
Employed	15 (6.1%)	136 (17.4%)	151 (14.7%)
Student	135 (54.7%)	128 (16.3%)	263 (25.5%)
Other	3 (1.2%)	8 (1.0%)	11 (1.1%)
Socio-economic status			
High	71 (28.7%)	201 (25.6%)	272 (26.4%)
Medium	94 (38.1%)	347 (44.3%)	441 (42.8%)
Low	82 (33.2%)	236 (30.1%)	318 (30.8%)
Food security			
Insecure	50 (20.2%)	125 (15.9%)	175 (17.0%)
Secure	197 (79.8%)	659 (84.1%)	856 (83.0%)

### Male condom use at last sex

#### Male condom use by race

The self-reported use of a male condom at last sex was 57.9% (595/1031) overall: 68.4% (169/247) among those aged 16–19 years and 54.6% (426/781) among those aged 20–24 years (*p* < 0.001) (see Additional file 1: Table S1). This was significantly higher among black women (59.6%, 538/903) compared to coloured women (43.4%, 49/113) (*p* = 0.001) (see Additional file 1: Table S1 and Additional file 2: Figure S1).

#### Male condom use by settlement type

Urban formal dwellers reported higher male condom use at last sex (61.2%, 207/338) relative to tribal settlements [the area within the rural settlement predominantly occupied by people of a common tribe] (49.7%, 75/151) (*p* = 0.017) (see Additional file 1: Table S1 and Additional file 3: Figure S2).

#### Male condom use by marital status

Married women (23.0%, 14/61) were less likely to report male condom use than those who were cohabitating (42.9%, 60/140) (*p* = 0.007) (see Additional file 1: Table S1 and Additional file 4: Figure S3).

#### Male condom use by level of education and employment status

Those who had matric (62.7%, 279/445) were more likely to report male condom use at last sex than those who only had primary education (47.2%, 17/36), although the difference was not statistically significant (*p* = 0.066) (see Additional file 1: Table S1 and Additional file 5: Figure S4). Women who were employed (51.7%, 78/151) were less likely to report male condom use compared to students (70.0%, 184/263) (*p* < 0.001) (see Additional file 1: Table S1 and additional file 6: Figure S5).

#### Male condom use by province and HIV status

**Fig. 1:**
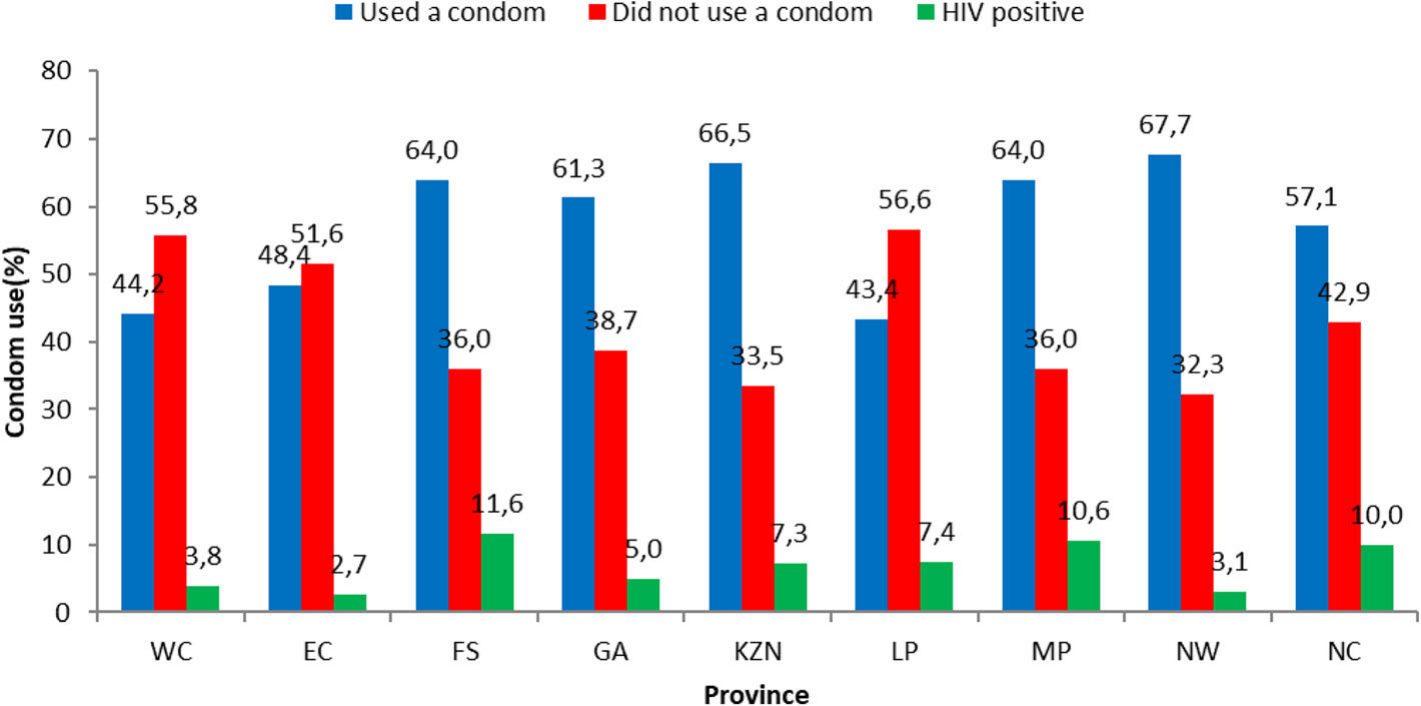
Male condom use at last sex by province among the sexually active young women aged 16–24 years, National HIV Communication Survey, South Africa, 2012

Of the nine provinces in South Africa, young women in the North West reported the highest rate of male condom use (67.7%, 44/65, 95% CI: 54.9–78.8) with a self-reported HIV infection of 3.1% (1/32, 95% CI: 0.08–16.2). Those in Limpopo had lowest self-reported male condom use (43.4%, 43/99, 95% CI: 33.5–53.8) which translated to self-reported HIV infection of 7.4% (4/54, 95% CI: 2.1–17.9). Self-reported male condom use at last sex was high in KwaZulu-Natal (66.5%, 135/203, 95% CI: 59.6–73.0); Mpumalanga (64.0%, 73/114, 95% CI: 54.5–72.8); Free State (64.0%, 57/89, 95% CI: 53.2–73.9), Gauteng (61.3%, 122/199, 95% CI: 54.2–68.1), and Northern Cape (57.1%, 12/21, 95% CI: 34.0–78.2). However, this did not translate to lower self-reported HIV infection [KwaZulu-Natal (7.3%, 11/151, 95% CI: 3.7–12.7), Mpumalanga (10.6%, 7/66, 95% CI: 4.4–20.6), Free State (11.6%, 8/69, 95% CI: 5.1–21.6), Gauteng (5.0%, 7/139, 95% CI: 2.0–10.1), and Northern Cape (10.0%, 1/10, 95% CI:0.3–44.5)]. Although young women in Eastern Cape (48.4%, 44/91, 95% CI: 37.7–59.1) and Western Cape (44.2%, 65/147, 95% CI: 36.0–52.6) had low self-reported condom use at last sex, self-reported HIV infection was also low [Eastern Cape (2.7%, 2/75, 95% CI: 0.3–9.3), Western Cape (3.8%, 5/131, 95% CI: 1.3–8.7)] (Fig. 1).

#### Male condom use and sexual behavioural practices among sexually active young women

The median age at sexual debut was 17 years (IQR 16–18 years). Young women who reported sexual debut at age 20 years or older (64.4%, 74/115) were more likely to have used male condom at the last sex compared to those who reported sexual debut at 19 years or younger (18–19 years [61.7%], 16–17 years [57.5%] and 0–15 years [46.2%], *p* = 0.005) (see Additional file 7: Table S2). Young women were more likely to use male condoms with a recent acquaintance (78.3%, 18/23) than with their main partner (61.0%, 424/695) (*p* < 0.001). Young women who reported living in same house with their partners (33.1%, 51/154) were less likely to self-report male condom use compared to women who reported to have their partners living in different province (63.9%, 46/72), same province (61.7%), same town (64.9%), or same neighborhood (59.9%) (*p* < 0.001) (see Additional file 7: Table S2).

#### Male condom use by sexual behavioral practices

**Fig. 2:**
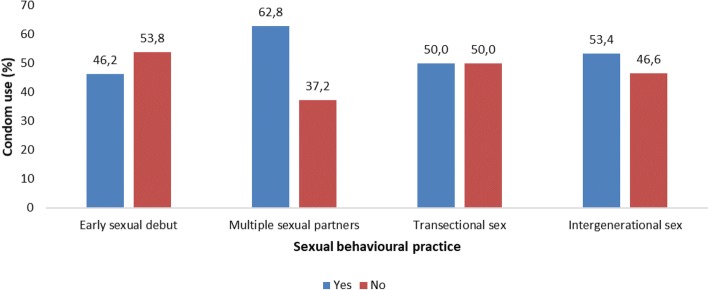
Male condom use at last sex by sexual behavioral practices among the sexually active young women aged 16–24 years, National HIV Communication Survey, South Africa, 2012

Young women who practiced intergenerational sex (sexual partnership between a young woman (15–24 years) and a man 5 or more years older) were more likely to use male condoms (53.4%, 222/416, 95% CI: 48.4–58.2) than those who did not (39.1%, 239/612) (*p* = 0.016) (Fig. 2) (see Additional file 7: Table S2).

#### Male condom use and HIV communication programs among sexually active young women

Television (TV) and radio were the most popular forms of media, with 83.5% (861/1031, 95% CI: 81.1–85.7) of young women watching TV and 69.2% (713/1031, 95% CI: 66.2–72.0) listening to radio more often (see Additional file 8: Figure S6). Additionally, women who watched TV daily (59.3%, 508/857, 95% CI: 55.9–62.6, *p* = 0.042); those who had daily access to internet (67.7%, 233/344, 95% CI: 62.5–72.6, *p* < 0.001) and those who were exposed to radio and televised HIV communication programmes such as “Intersexions” (63.3%, 368/581, 95% CI: 59.3–67.3, p < 0.001) and “Brothers for Life” (63.4%, 357/563, 95% CI: 59.3–67.4, *p* < 0.001) in the past 12 months were more likely to use a condom at last sexual encounter (see Additional file 9: Table S3).

#### Factors associated with male condom use at last sex among sexually active young women

After adjusting for potential confounding in the multivariable analysis delayed sexual debut (Adjusted Odds Ratio [aOR] 2.1, 95% CI: 1.2 to 3.7, *p* = 0.006); living in a different province from a regular sexual partner (aOR 2.4, 95% CI: 1.2 to 4.8, *p* = 0.017); being a student (aOR 1.6, 95% CI: 1.2 to 2.3, *p* = 0.005); having sex with a recent acquaintance compared with a main partner (aOR 8.1, 95% CI: 2.2 to 29.8, *p* = 0.002); having access to internet (aOR 1.5, 95% CI: 1.1 to 2.1, p = 0.006) and exposure to HIV communication programmes such as “Intersexions” (aOR 1.3, 95% CI: 1.0 to 1.7, *p* = 0.056) and “i-Life” (aOR 3.1, 95% CI: 1.2 to 8.6, *p* = 0.025) were significantly associated with use of male condom at last sex (Table 2).

**Table 2:** Factors associated with male condom use among sexually active young women aged 16–24 years, National HIV Communication Survey, South Africa, 2012

Variable	Condom use at last sex N	Non-condom use at last sex N	Bivariate analysis			Multivariable analysis		
			Crude OR	95% CI	*p*-value	Adjusted OR	95% CI	*p*-value
Age group								
16–19 years	169	78	1	Reference	Reference	_	_	_
20–24 years	426	355	0.6	0.4–0.7	< 0.001	_	_	_
Race								
White	8	4	1	Reference	Reference	_	_	_
Black	538	365	0.7	0.2–2.5	< 0.001	_	_	_
Coloured	49	64	0.4	0.1–1.3	0.001			
Marital status								
Married	14	47	1	Reference	Reference	_	_	_
Single	265	164	5.7	3.0–10.6	0.735	_	_	_
Stable relationship	243	136	6.3	3.3–11.7	0.682	_	_	_
Cohabitation	60	80	2.6	1.3–5.2	0.84	_	_	_
Settlement type								
Farming settlement	12	11	1	Reference	Reference	_	_	_
Urban formal	207	131	1.7	0.7–4.0	< 0.001	_	_	_
Urban informal	232	159	1.6	0.7–3.7	0.6	_	_	_
Peri-urban	69	56	1.3	0.6–3.3	0.24	_	_	_
Tribal settlement	75	76	1.1	0.4–2.6	0.017	_	_	_
Employment status								
Unemployed	327	277	1	Reference	Reference	1	Reference	Reference
Employed	78	72	0.9	0.6–1.3	0.638	0.8	0.5–1.2	0.242
Student	184	79	2.0	1.4–2.7	< 0.001	1.6	1.2–2.3	0.005
Level of Education								
Primary	17	19	1	Reference	Reference	_	_	_
Grade 11	245	209	1.3	0.7–2.6	0.436	_	_	_
Matric	279	165	1.9	1.0–3.7	0.067	_	_	_
Tertiary	54	37	1.6	0.8–3.5	0.217	_	_	_
Multiple sexual partners^a^								
No	536	398	1	Reference	Reference	_	_	_
Yes	59	35	1.3	0.8–1.9	0.315	_	_	_
Sexual debut								
0–15 years	73	85	1	Reference	Reference	1	Reference	Reference
16–17 years	248	183	1.6	1.1–2.3	0.015	1.4	1.0–2.1	0.072
18–19 years	200	124	1.9	1.3–2.8	0.001	1.8	1.2–2.7	0.008
20–24 years	74	41	2.1	1.3–3.4	0.003	2.1	1.2–3.7	0.006
Transactional sex^a^								
No	547	387	1	Reference	Reference	_	_	_
Yes	48	46	0.7	0.5–1.1	0.162	_	_	_
Intergenerational sex^a^								
No	373	239	1	Reference	Reference	_	_	_
Yes	222	194	0.7	0.6–0.9	0.016	_	_	_
Living separately from a regular partner								
Same house	51	103	1	Reference	Reference	1	Reference	Reference
Same neighborhood	188	128	3.0	2.0–4.4	< 0.001	1.8	1.0–3.2	0.038
Same town or area	213	115	3.7	2.5–5.6	< 0.001	2.2	1.2–3.9	0.006
Same province	92	57	3.3	2.0–5.2	< 0.001	1.8	1.0–3.3	0.071
Different province	46	26	3.6	2.0–6.4	< 0.001	2.4	1.2–4.8	0.017
Relationship type								
Main partner	424	275	1	Reference	Reference	1	Reference	Reference
A friend	39	23	1.1	0.6–1.9	< 0.001	4.5	1.8–11.7	0.002
Someone known for while	58	27	1.4	0.9–2.3	< 0.001	5.3	2.1–13.5	< 0.001
Recent acquaintance	18	5	2.3	0.9–6.4	< 0.001	8.1	2.2–29.8	0.002
Radio								
Never listened^a^	171	145	1	Reference	Reference	_	_	_
Listened¥	424	288	1.2	1.0. - 1.6	0.104	_	_	_
TV								
Never watched^a^	87	84	1	Reference	Reference	_	_	_
Watched^a^	508	349	1.4	1.0–2.0	0.043	_	_	_
Magazine								
Never read^a^	347	258	1	Reference	Reference	_	_	_
Read^a^	248	175	1.1	0.8–1.4	0.684	_	_	_
Newspaper								
Never read^a^	268	188	1	Reference	Reference	_	_	_
Read¥	327	245	0.9	0.7–1.2	0.605	_	_	_
Internet								
Had no access^a^	362	322	1	Reference	Reference	1	Reference	Reference
Had access^a^	233	111	1.9	1.4–2.4	< 0.001	1.5	1.1–2.1	0.006
Intersexions^*(TV programme)*^								
Not exposed^a^	227	220	1	Reference	Reference	1	Reference	Reference
Exposed^a^	368	213	1.7	1.3–2.2	< 0.001	1.3	1.0–1.7	0.056
4Play:Sex Tips for Girls^*(TV programme)*^								
Not exposed^a^	467	337	1	Reference	Reference	_	_	_
Exposed^a^	128	96	1.0	0.7–1.3	0.801	_	_	_
Brothers for Life^*(TV programme)*^								
Not exposed^a^	238	227	1	Reference	Reference	_	_	_
Exposed^a^	357	206	1.7	1.3–2.1	< 0.001	_	_	_
Scrutinize^*(TV programme)*^								
Not exposed^a^	106	106	1	Reference	Reference	_	_	_
Exposed^a^	489	327	1.5	1.1–2.0	0.009	_	_	_
Soul City^*(TV programme)*^								
Not exposed^a^	240	192	1	Reference	Reference	_	_	_
Exposed^a^	355	241	1.2	0.9–1.5	0.199	_	_	_
loveLife^*(TV and radio programme)*^								
Not exposed^a^	560	412	1	Reference	Reference	_	_	_
Exposed^a^	35	21	1.2	0.7–2.1	0.472	_	_	_
Siyanqoba Beat it! ^*(TV programme)*^								
Not exposed^a^	247	234	1	Reference	Reference	_	_	_
Exposed^a^	348	199	1.7	1.3–2.1	< 0.001	_	_	_
We BEAT TB^*(TV programme)*^								
Not exposed^a^	188	182	1	Reference	Reference	_	_	_
Exposed^a^	407	251	1.6	1.2–2.0	0.001	_	_	_
i-Life^*(Radio programme)*^								
Not exposed^a^	571	426	1	Reference	Reference	1	Reference	Reference
Exposed¥	24	7	2.6	1.1–6.0	0.031	3.1	1.2–8.6	0.025

^a^In the last 12 months

#### Self-reported HIV infection among sexually active young women

The proportion of self-reported HIV infection was 6.3% (46/724, 95% CI: 4.7–8.4) among sexually active young women: 3.1% (5/161, 95% CI: 1.0–7.1) among those aged 16–19 years and 7.2% (41/566, 95% CI: 5.2–9.7) among those aged 20–24 years (*p* = 0.057). Self-reported HIV infection was significantly higher among young women who reported multiple sexual partnerships in the past 12 months (18.8%, 12/64, 95% CI: 10.1–30.5, *p* < 0.001); early sexual debut (15.3%, 17/111, 95% CI: 9.2–23.4, *p* = 0.012); intergenerational sex (9.2%, 27/295, 95% CI: 6.1–13.0, *p* = 0.010) and among those who reported transactional sex (14.6%, 8/55, 95% CI: 6.5–26.7, *p* = 0.009) (see Additional file 10: Table S4).

#### Socio-demographic and sexual behavioral practices predicting HIV infection among sexually active young women

The strongest predictors of self-reported HIV infection in a multivariable analysis were reported practice of multiple sexual partnerships (aOR 2.9, 95% CI: 1.2–7.0, *p* = 0.017), intergenerational sex (aOR 2.0, 95% CI: 1.0–4.0, *p* = 0.047), living in Mpumalanga (aOR 8.3, CI: 1.4–51.0, *p* = 0.022) and being aged 20 to 24 years (OR 7.3, 95% CI: 2.3–23.1, *p* = 0.001) (Table 3). The odds of self-reported HIV infection decreased with delayed sexual debut (aOR 0.2, 95% CI: 0.03–0.70, *p* = 0.014), food security (aOR 0.3, 95% CI: 0.2–0.7, *p* = 0.007), tertiary education (aOR 0.04, 95% CI: 0.003–0.510, *p* = 0.013) and cohabitating (aOR 0.2, 95% CI: 0.1–0.9, *p* = 0.028) (Table 3).

**Table 3:** Demographic and sexual behavioral practices predicting HIV infection among sexually active young women aged 16–24 years, National HIV Communication Survey, South Africa, 2012

Variable	HIV + ve		Univariate analysis			Multivariable analysis		
	N	%	Crude OR	95% CI	p-value	Adjusted OR	95% CI	*p*-value
Age group								
16–19 years	5	3.1	1	Reference	Reference	1	Reference	Reference
20–24 years	41	7.2	2.4	0.9–6.3	0.065	7.3	2.3–23.1	0.001
Province								
Eastern Cape	2	2.7	1	Reference	Reference	1	Reference	Reference
Free State	8	11.6	4.8	1.0–23.4	0.053	4.2	0.8–28.9	0.085
Gauteng	7	5.0	1.9	0.4–9.6	0.418	4.0	0.7–23.7	0.125
KwaZulu-Natal	11	7.3	2.9	0.6–13.3	0.178	5.4	1.0–30.0	0.056
Limpopo	4	7.4	2.9	0.5–16.6	0.226	4.1	0.6–29.5	0.166
Mpumalanga	7	10.6	4.3	0.9–21.6	0.074	8.3	1.4–51.0	0.022
North West	1	3.1	1.2	0.1–13.5	0.895	1.5	0.1–22.6	0.768
Northern Cape	1	10.0	4.1	0.3–49.3	0.272	4.6	0.3–83.5	0.300
Western Cape	5	3.8	1.4	0.3–7.7	0.663	2.5	0.4–16.0	0.323
Settlement type								
Urban formal	11	4.6	1	Reference	Reference	_	_	_
Urban informal	24	8.0	1.8	0.9–3.8	0.111	_	_	_
Peri-urban	7	8.3	1.9	0.7–5.1	0.200	_	_	_
Tribal settlement	3	3.4	0.7	0.2–2.7	0.634	_	_	_
Farming	1	7.7	1.7	0.2–14.6	0.609	_	_	_
Housing type								
Formal house	7	3.4	1	Reference	Reference	_	_	_
Mostly formal	16	8.3	2.5	1.0–6.3	0.045	_	_	_
Mostly informal	16	8.7	2.7	1.1–6.6	0.035	_	_	_
Squatter camp	2	2.8	0.8	0.2–4.0	0.802	_	_	_
Traditional house	5	7.9	2.4	0.7–7.9	0.142	_	_	_
Level of Education								
Primary	4	26.7	1	Reference	Reference	1	Reference	Reference
Grade 11	27	8.0	0.2	0.1–0.8	0.020	0.2	0.03–0.73	0.017
Matric	14	4.6	0.1	< 0.0–0.5	0.002	0.1	0.02–0.56	0.007
Tertiary	1	1.4	< 0.01	< 0.01–0.4	0.006	0.04	< 0.01–0.51	0.013
Marital status								
Single	26	7.7	1	Reference	Reference	1	Reference	Reference
Stable relationship	11	4.5	0.6	0.3–1.2	0.125	0.5	0.2–1.3	0.156
Cohabitation	4	4.4	0.5	0.2–1.6	0.278	0.2	0.1–0.9	0.028
Married	5	9.8	1.3	0.5–3.6	0.609	0.8	0.2–3.6	0.798
Employment status								
Unemployed	31	7.0	1	Reference	Reference	_	_	_
Employed	8	8.1	1.2	0.5–2.6	0.693	_	_	_
Student	7	4.0	0.6	0.2–1.3	0.169	_	_	_
Socio-economic status								
High	7	3.7	1	Reference	Reference	_	_	_
Medium	25	8.0	2.2	0.9–5.3	0.066	_	_	_
Low	14	6.2	1.7	0.7–4.3	0.255	_	_	_
Food security¥								
Insecure	17	14.2	1	Reference	Reference	1	Reference	Reference
Secured	29	4.8	0.3	0.2–0.6	< 0.001	0.3	0.2–0.7	0.007
Multiple sexual partners¥								
No	34	5.1	1	Reference	Reference	1	Reference	Reference
Yes	12	18.8	4.3	2.1–8.7	< 0.001	2.9	1.2–7.0	0.017
Sexual debut								
0–15 years	17	15.3	1	Reference	Reference	1	Reference	Reference
16–17 years	19	6.4	0.4	0.2–0.8	0.006	0.3	0.1–0.7	0.005
18–19 years	7	2.9	0.2	0.1–0.4	< 0.001	0.1	0.03–0.31	< 0.001
20–24 years	3	3.9	0.2	0.1–0.8	0.019	0.2	0.03–0.70	0.014
Transactional sex¥								
No	38	5.7	1	Reference	Reference	1	Reference	Reference
Yes	8	14.6	2.8	1.3–6.4	0.012	1.8	0.6–4.6	0.313
Intergenerational sex¥								
No	19	4.4	1	Reference	Reference	1	Reference	Reference
Yes	27	9.1	2.2	1.2–4.0	0.011	2.0	1.0–4.0	0.047

_ Not statistically significant

¥In the last 12 months

## Discussion

This study described the prevalence of male condom use and determined the associated socio-demographic factors and sexual behaviours among sexually active women aged 16–24 years in South Africa.

The overall prevalence of male condom use at last sex was just less than 60%. Delayed sexual debut, living separately from a regular sexual partner and having sex with a recent acquaintance were significantly associated with condom use at last sex as independent variables. We also determined that being a student was the only socio-demographic factor associated with condom use at last sex among young women. Identifying socio-demographic factors and sexual behavioural practices predicting condom use in this population group is crucial to identify gaps and inform HIV prevention programmes to mitigate HIV risk factors.

The prevalence of condom use reported in this study is similar to the prevalence of condom use (58.4%) reported among youths aged 15–24 years in the 2012 population-based HIV survey conducted by Human Science Research Council (HSRC) in South Africa [20]. Furthermore, evidence from previous HSRC surveys suggests that the prevalence of condom use increased from 46 to 73% between 2002 and 2008 among young women [20]. This increase coincided with heightened mass media campaigns and behavioural change programmes implemented between 1999 and 2009, including the National Department of Health’s Khomanani Campaign [21–23]. Khomanani, a televised programme from 2001 to 2008, was based on behavioural change communication theory aimed at reducing new HIV infections by increasing personal risk perception and advocating for safer sex practices such as condom use [21–23]. The observed decline coincides with the absence of sustained mass media programmes such as Khomanani [21–23].

The odds of using a male condom were three times higher among women who had a late sexual debut than those with early sexual debut, suggesting that condom use was significantly associated with delayed sexual debut. This is consistent with findings from many studies conducted in different sub-Saharan African countries [14–17]. Delaying the age at which young women first have sex is a critical factor in reducing HIV exposure among young women. Behavioral interventions encouraging later sexual debut and encouraging condom use at first sexual encounter might increase condom usage among young women in South Africa.

The geographic distance between sexual partners was associated with condom use among sexually active young women. As a result, young women were more likely to use a condom at last sex if they were living separately from their regular partners. These findings were consistent with the findings of a study conducted in rural KwaZulu-Natal among sexually active young adults [9]. The possible explanation to this could be lack of trust and unfaithfulness between sexual partners emphasizing the importance of developing HIV prevention interventions that address HIV risk factors at a structural and partner level [9, 17].

The type of relationship plays a role in the decision making process regarding condom use among sexual partners [11]. In this study, relationship type was found to be a strong predictor of condom use among sexually active young women. Sexually active young women were five times more likely to use a condom with an acquaintance than with a regular partner. This finding is consistent with findings by Prata et al. [11] and Matseke et al. [24] in a studies conducted in Angola and South Africa respectively. They also found that condom use at last sexual encounter was significantly higher among those who practiced casual sex compared to those who had sex with a main sexual partner (21%) [11, 24]. This could indicate that young women perceived casual sex as a greater risk for HIV infection, hence the need to use condoms. Sex education programmes should develop specific messages targeting women who engage in casual sex in order to increase their risk perception and broaden their knowledge.

Students were two times more likely to use condoms at last sex than those who were unemployed suggesting that being a student was significantly associated with male condom use at last sexual encounter. This could also explain the higher prevalence of HIV among unemployed youths and those who are not in school. National HIV prevention programmes should not only adopt the school-based strategy to reduce the proportion of young people engaging in unprotected sex, but they should also focus on reaching those young women who are out of school.

### Study limitations

This study may have been affected by recall bias commonly found in data from cross-sectional surveys. This may have affected the ability to recall whether a condom was used or not during the last sexual encounter among study participants leading to underestimation or overestimation of the study findings. Similarly, respondents who had a recent sexual encounter were more likely to report condom use than those who had sex a few months prior to the survey, resulting in an under-estimation of the prevalence of condom use. Furthermore, the recall of age at sexual debut, especially for those aged 20–24 years, might have contributed to some reporting bias. Additionally, social desirability is likely to occur in settings where there is extensive publicity in favor of condom use. This may lead to over-estimation of reported condom use due to a desire to appear to hold contemporary views and approve of use of condoms by study participants. The association of program exposure, and the inferences about the absence or presence of programs with increases and declines in condom use are subject to a confluence of other intermediary factors and HIV programs that were not measured in this study. The Stata regression analysis procedure automatically removes variables that are collinear. However, although there was no multi-collinearity detected, this may have affected the association between HIV programme exposure and condom at last sex resulting to underestimation or overestimation of measure of association.

## Conclusions

This study found that male condom use at last sexual encounter among sexually active young women was approximately 60%. Male condom use was associated with delayed sexual debut, living separately from a regular partner, being a student compared to being unemployed, having sex with a recent acquaintance as compared with a regular partner, having access to media channels such as the internet, and exposure to HIV communication programmes such as Intersexions in the past 12 months. Delayed sexual debut, being food secure, having tertiary education and cohabitation were significantly protective of HIV infection. The practice of multiple sexual partnerships, intergenerational sex, living in Mpumalanga province and being aged 20–24 years were significantly associated with HIV infection among young women. Young women out of school reported much lower levels of condom use. The prevalence of condom use and risky sexual behaviours varied considerably across various geographic locations within the nine provinces of South Africa. Young women at risk for HIV infection are a heterogeneous group and need different messaging to encourage consistent condom use. Although there was increased condom use at last sexual encounter among those who engaged in multiple sexual partnerships in this study, there is a great need to discourage the practice of multiple sexual partnerships and advocate for consistent condom use in all relationships with all sexual partners. Additionally, sustained behavioural change interventions aimed at condom messaging and marketing campaigns at a national level are needed to promote condom use among young women. Furthermore, the existing behavioural intervention programmes and HIV communication programmes should encourage the delay of sexual initiation and condom usage among young women. HIV prevention interventions should seek to address HIV risk factors at a structural and partner level. Targeted interventions aimed at addressing the structural and economic drivers of HIV, such as food insecurity, are needed. Targeted interventions are also needed to reduce the vulnerability of young women to HIV infection by retaining them in schools as well as increasing their access to tertiary education. Additionally, national HIV prevention programmes should not only adopt the school-based strategy to reduce the proportion of young people engaging in unprotected sex but they should also focus on reaching those young girls who are out of school. Communication programmes and mass media campaigns should strongly encourage consistent condom use and discourage the practice of multiple sexual partnerships and intergenerational sex among young women.

## Additional Files

Additional File 1: **Table S1.** Male condom use at last sex by socio-demographic factors among sexually active young women aged 16–24 years, National HIV Communication Survey, South Africa, 2012, Frequency distribution table with bivariate analysis of socio-demographic factors associated with male condom use at last sex among Sexually Active Young Women in South Africa with percentages and Chi-square Inferences, Socio-demographic factors associated with condom use. (DOCX 14 kb)

Additional File 2: **Figure S1.** Male condom use at last sex by race among the sexually active young women aged 16–24 years, National HIV Communication Survey, South Africa, 2012. Graphical presentation of male condom use by race using percentages, Male condom use by race. (DOCX 28 kb)

Additional File 3: **Figure S2.** Male condom use at last sex by settlement type among the sexually active young women aged 16–24 years, National HIV Communication Survey, South Africa, 2012. Graphical presentation of male condom use by settlement type using percentages, Male condom use by settlement type. (DOCX 35 kb)

Additional File 4: **Figure S3.** Male condom use at last sex by marital status among the sexually active young women aged 16–24 years, National HIV Communication Survey, South Africa, 2012**,** Graphical presentation of male condom use by marital status using percentages, Male condom use by marital status. (DOCX 32 kb)

Additional File 5: **Figure S4.** Male condom use at last sex by level of education among the sexually active young women aged 16–24 years, National HIV Communication Survey, South Africa, 2012**,** Graphical presentation of male condom use by level of education using percentages, Male condom use by level of education. (DOCX 32 kb)

Additional File 6: **Figure S5.** Male condom use at last sex by employment status among the sexually active young women aged 16–24 years, National HIV Communication Survey, South Africa, 2012**,** Graphical presentation of male condom use by employment status using percentages, Male condom use by employment status. (DOCX 28 kb)

Additional File 7: **Table S2.** Sexual behavioural practices and condom use at last sex among the sexually active young women aged 16–24 years, National HIV Communication Survey, South Africa, 2012**,** Frequency distribution table with bivariate analysis of Sexual behavioural practices associated with male condom use at last sex among Sexually Active Young Women in South Africa with percentages and Chi-square Inferences. Sexual behavioural practices associated with condom use. (DOCX 14 kb)

Additional File 8: **Figure S6.** Media sources accessed by young women aged 16–24 years, National HIV Communication Survey, South Africa, 2012**,** Graphical presentation of media sources accessed by young women using percentages, Media sources. (DOCX 28 kb)

Additional File 9: **Table S3.** Access to media and exposure to HIV Communication Programmes among young women aged 16–24 years, National HIV Communication Survey, South Africa, 2012**,** Frequency distribution table showing Access to media and exposure to HIV Communication Programmes among Sexually Active Young Women in South Africa with percentages and Chi-square Inferences, Access to media and exposure to HIV Communication Programmes (DOCX 16 kb)

Additional File 10: **Table S4.** Demographic and sexual behaviours determining HIV infection among sexually active young women aged 16–24 years, National HIV Communication Survey, South Africa, 2012, Factors associated with HIV Infection among Sexually Active Young Women in South Africa with percentages and Chi-square Inferences, Determinants of HIV Infection. (DOCX 15 kb)

## Abbreviations

AIDS: Acquired immunodeficiency syndrome
aOR: Adjusted odd ratio
ARVs: Anti-retrovirals
CI: Confidence interval
FGI: Freshly ground insights
HDA: Health and development africa
HIV: Human immunodeficiency virus
HSRC: Human science research council
JCRC: Joint clinical research
JHHESA: Johns hopkins health and education in south africa
JHU-CCP: Johns hopkins bloomberg school of public health center for communication programs
NCS: Third national HIV communication survey
OR: Odds ratios
PEPFAR: United states president’s emergency plan for AIDS relief
SAFETP: South african field epidemiology training program
TV: Television (TV)
UNAIDS: Joint united nations programme on HIV/AIDS
USAID: United states agency for international development
WHO: World Health Organization
