# Supplementary material for: Predictors of male condom use among sexually active heterosexual young women in South Africa, 2012
Source: BMC Public Health. 2018 Sep 24;18:1137. doi: 10.1186/s12889-018-6039-8 (PMC6154873; doi:10.1186/s12889-018-6039-8)
Supplement: Supplementary file 1 — Table S1. Male condom use at last sex by socio-demographic factors among sexually active young women aged 16–24 years, National HIV Communication Survey, South Africa, 2012, Frequency distribution table with bivariate analysis of socio-demographic factors associated with male condom use at last sex among Sexually Active Young Women in South Africa with percentages and Chi-square Inferences, Socio-demographic factors associated with condom use. (DOCX 14 kb) [file 12889_2018_6039_MOESM1_ESM.docx]

| ***Variable*** | ***Condom use at last sex*** | | ***Non-condom use at last sex*** | | ***P-value*** |
| --- | --- | --- | --- | --- | --- |
|  | ***N (%)*** | ***95% CI*** | ***N (%)*** | ***95% CI*** |  |
| **Overall condom use** | **595 (57.9%)** | **54.6 - 60.7** | **433 (42.1%)** | **39.1 - 45.2** |  |
| ***Age group* (3/1,031)*** |  |  |  |  | ***<0.001*** |
| 16–19 years | 169 (68.4%) | 62.2 - 74.2 | 78 (31.6%) | 25.8 - 37.8 |  |
| 20–24 years | 426 (54.6%) | 51.0 - 58.1 | 355 (45.4%) | 41.9 - 49.0 |  |
| ***Settlement type*(3/1,031)*** |  |  |  |  | ***0.149*** |
| Farming settlement | 12 (52.1%) | 30.6 - 73.2 | 11 (47.8%) | 26.8 - 69.4 |  |
| Urban formal | 207 (61.2%) | 55.8 - 66.5 | 131 (38.8%) | 33.5 - 44.2 |  |
| Urban informal | 232 (59.3%) | 54.3 - 64.2 | 159 (40.7%) | 35.8 - 45.7 |  |
| Peri-urban | 69 (55.2%) | 46.0 - 64.1 | 56 (44.8%) | 36.0 - 54.0 |  |
| Tribal settlement | 75 (49.7%) | 41.4 - 57.9 | 76 (50.3%) | 42.1 - 58.6 |  |
| ***Marital status*(22/1,031)*** |  |  |  |  | ***<0.001*** |
| Married | 14 (23.0%) | 13.2 - 35.5 | 47 (67.0%) | 64.5 - 86.4 |  |
| Single | 265 (61.8%) | 57.0 - 66.4 | 164 (38.2%) | 33.6 - 43.0 |  |
| Stable relationship | 243 (64.1%) | 59.1 - 69.9 | 136 (35.9%) | 31.0 - 40.9 |  |
| Cohabitation | 60 (42.9%) | 34.5 - 51.5 | 80 (57.1%) | 48.5 - 65.5 |  |
| ***Level of Education*(6/1,031)*** |  |  |  |  | ***0.019*** |
| Primary | 17 (47.2%) | 30.4 - 64.5 | 19 (52.8%) | 35.5 - 69.6 |  |
| Grade 11 | 245 (54.0%) | 49.3 - 58.6 | 209 (46.0%) | 41.4 - 50.7 |  |
| Matric | 279 (62.8%) | 58.1 - 67.3 | 165 (37.2%) | 32.7 - 41.8 |  |
| Tertiary | 54 (59.3%) | 48.5 - 69.5 | 37 (40.7%) | 30.5 - 51.5 |  |
| ***Employment status*(14/1,031)*** |  |  |  |  | ***<0.001*** |
| Unemployed | 327 (54.1%) | 50.1 - 58.2 | 277 (45.9%) | 41.8 - 49.9 |  |
| Employed | 78 (51.7%) | 43.7 - 60.2 | 72 (48.3%) | 39.8 - 56.3 |  |
| Student | 184 (70.0%) | 23.4 - 83.3 | 79 (30.0%) | 24.6 - 36.0 |  |
| ***Race*(3/1,031)*** |  |  |  |  | ***0.004*** |
| White | 8 (66.7%) | 34.9 - 90.1 | 4 (33.3%) | 9.9 - 65.1 |  |
| Coloured | 49 (43.4%) | 34.1 - 53.0 | 64 (56.6%) | 47.0 - 65.9 |  |
| Black | 538 (59.6%) | 56.3 - 62.8 | 365 (40.4%) | 37.2 - 43.7 |  |

***Missing data**
