# Supplementary figures and images for: Predictors of male condom use among sexually active heterosexual young women in South Africa, 2012
Source: BMC Public Health. 2018 Sep 24;18:1137. doi: 10.1186/s12889-018-6039-8 (PMC6154873; doi:10.1186/s12889-018-6039-8)

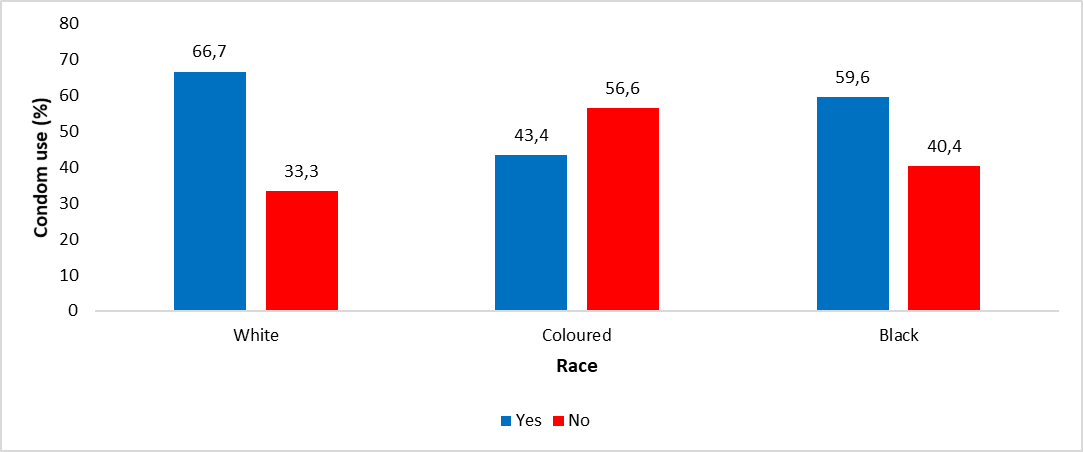

Supplement: Supplementary file 2 — Figure S1. Male condom use at last sex by race among the sexually active young women aged 16–24 years, National HIV Communication Survey, South Africa, 2012. Graphical presentation of male condom use by race using percentages, Male condom use by race. (DOCX 28 kb) [file 12889_2018_6039_MOESM2_ESM.docx]

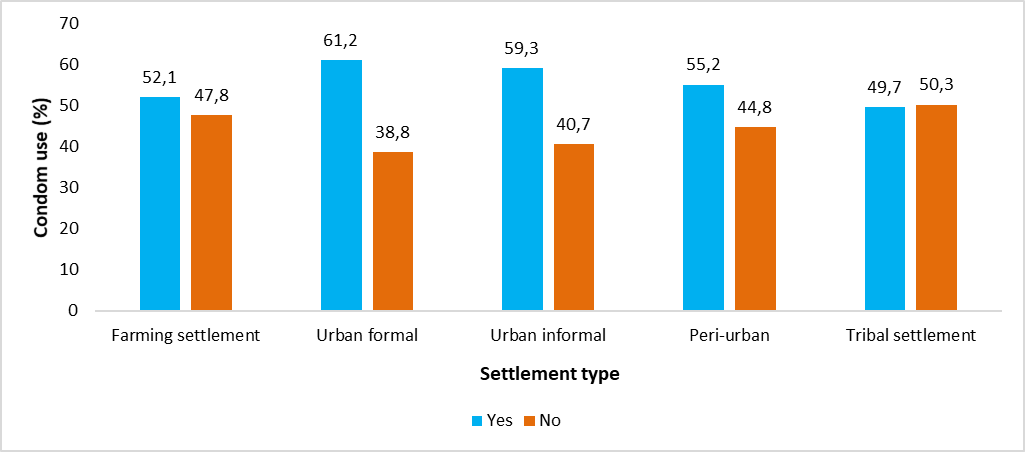

Supplement: Supplementary file 3 — Figure S2. Male condom use at last sex by settlement type among the sexually active young women aged 16–24 years, National HIV Communication Survey, South Africa, 2012. Graphical presentation of male condom use by settlement type using percentages, Male condom use by settlement type. (DOCX 35 kb) [file 12889_2018_6039_MOESM3_ESM.docx]

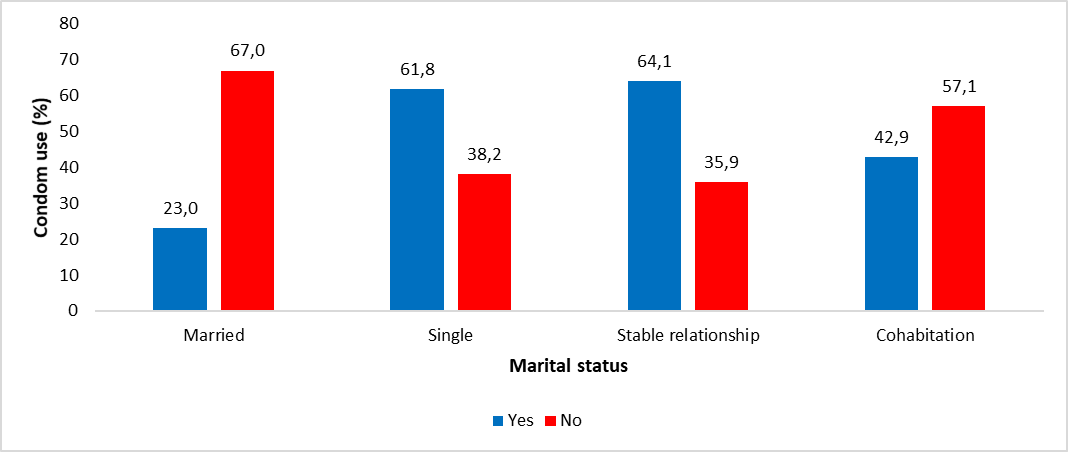

Supplement: Supplementary file 4 — Figure S3. Male condom use at last sex by marital status among the sexually active young women aged 16–24 years, National HIV Communication Survey, South Africa, 2012, Graphical presentation of male condom use by marital status using percentages, Male condom use by marital status. (DOCX 32 kb) [file 12889_2018_6039_MOESM4_ESM.docx]

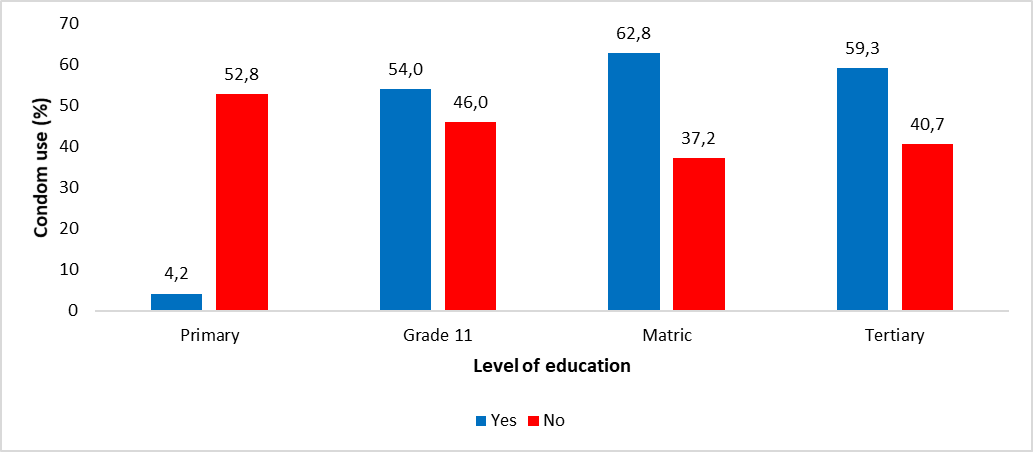

Supplement: Supplementary file 5 — Figure S4. Male condom use at last sex by level of education among the sexually active young women aged 16–24 years, National HIV Communication Survey, South Africa, 2012, Graphical presentation of male condom use by level of education using percentages, Male condom use by level of education. (DOCX 32 kb) [file 12889_2018_6039_MOESM5_ESM.docx]

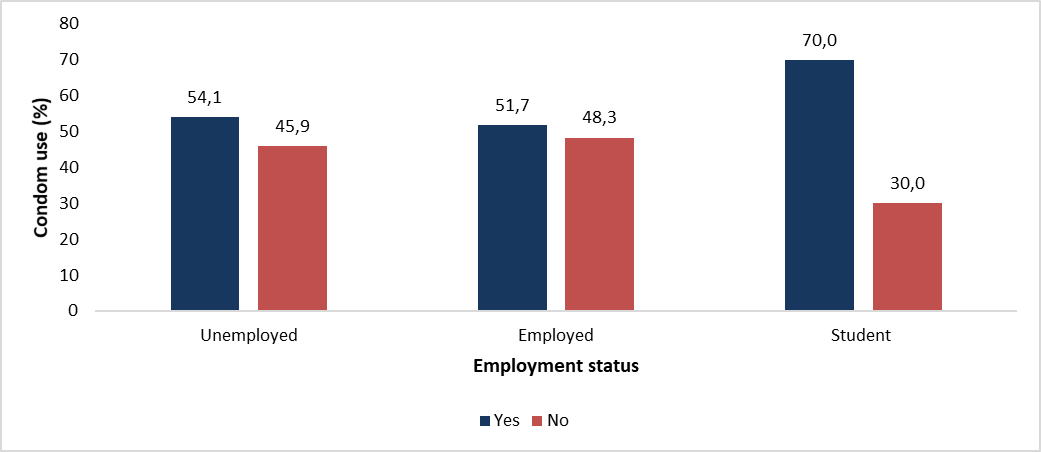

Supplement: Supplementary file 6 — Figure S5. Male condom use at last sex by employment status among the sexually active young women aged 16–24 years, National HIV Communication Survey, South Africa, 2012, Graphical presentation of male condom use by employment status using percentages, Male condom use by employment status. (DOCX 28 kb) [file 12889_2018_6039_MOESM6_ESM.docx]

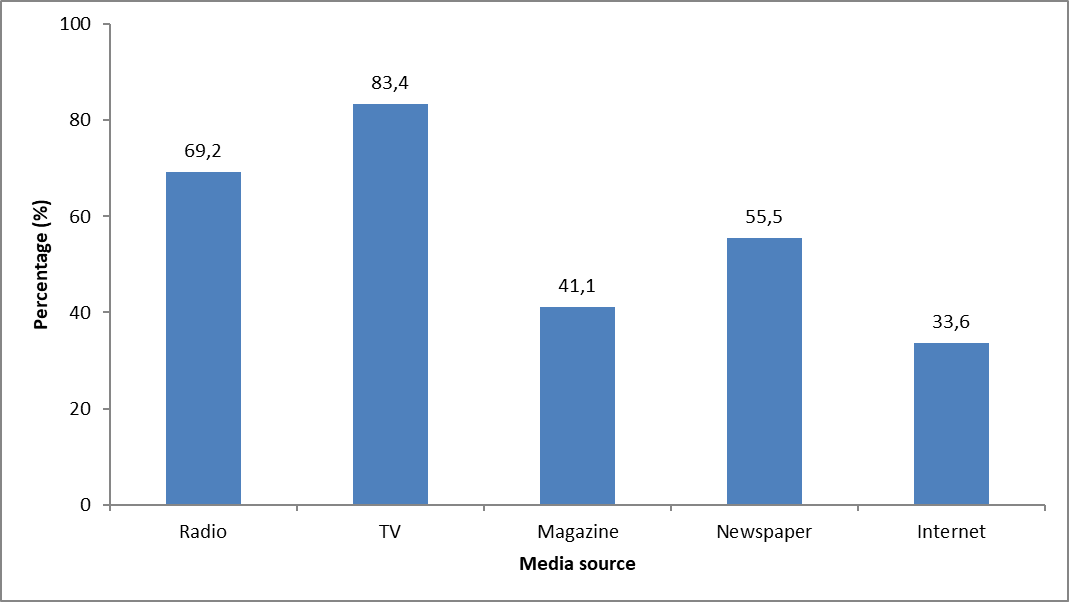

Supplement: Supplementary file 8 — Figure S6. Media sources accessed by young women aged 16–24 years, National HIV Communication Survey, South Africa, 2012, Graphical presentation of media sources accessed by young women using percentages, Media sources. (DOCX 28 kb) [file 12889_2018_6039_MOESM8_ESM.docx]
