# Supplementary material for: Predictors of male condom use among sexually active heterosexual young women in South Africa, 2012
Source: BMC Public Health. 2018 Sep 24;18:1137. doi: 10.1186/s12889-018-6039-8 (PMC6154873; doi:10.1186/s12889-018-6039-8)
Supplement: Supplementary file 7 — Table S2. Sexual behavioural practices and condom use at last sex among the sexually active young women aged 16–24 years, National HIV Communication Survey, South Africa, 2012, Frequency distribution table with bivariate analysis of Sexual behavioural practices associated with male condom use at last sex among Sexually Active Young Women in South Africa with percentages and Chi-square Inferences. Sexual behavioural practices associated with condom use. (DOCX 14 kb) [file 12889_2018_6039_MOESM7_ESM.docx]

| ***Variable*** | ***Condom use at last sex*** | | ***Non-condom use at last sex*** | | ***P-value (chi-square test)*** |
| --- | --- | --- | --- | --- | --- |
|  | ***N (%)*** | ***95% CI*** | ***N (%)*** | ***95% CI*** |  |
| ***Overall condom use*** | **595 (57.9%)** | **54.6 - 60.7** | **433 (42.1%)** | **39.1 - 45.2** |  |
| ***Age group* (3/1,031)*** |  |  |  |  | *<0.001* |
| 16–19 years | 169 (68.4%) | 62.2 - 74.2 | 78 (31.6%) | 25.8 - 37.8 |  |
| 20–24 years | 426 (54.6%) | 51.0 - 58.1 | 355 (45.4%) | 41.9 - 49.0 |  |
| ***Multiple sexual partners*(3/1,031) ¥*** |  |  |  |  | *0.314* |
| No | 536 (57.4%) | 54.1 - 60.6 | 398 (42.6%) | 39.4 - 45.9 |  |
| Yes | 59 (62.8%) | 52.2 - 72.5 | 35 (37.2%) | 27.5 - 47.8 |  |
| ***Early sexual debut*(3/1,031)*** |  |  |  |  | *0.005* |
| 0–15 years | 73 (46.2%) | 38.2 - 54.3 | 85 (53.8%) | 45.7 - 61.8 |  |
| 16–17 years | 248 (57.5%) | 52.5 - 62.3 | 183 (42.5%) | 37.7 - 47.3 |  |
| 18–19 years | 200 (61.7%) | 56.2 - 67.0 | 124 (38.3%) | 33.0 - 43.8 |  |
| 20–24 years | 74 (64.3%) | 54.9 - 73.1 | 41 (35.7%) | 26.9 - 45.1 |  |
| ***Relationship type*(162/1,031)*** |  |  |  |  | *0.209* |
| Main partner | 424 (60.9%) | 56.9 - 64.3 | 275 (39.1%) | 35.7 - 43.1 |  |
| A friend | 39 (62.9%) | 49.7 - 74.8 | 23 (37.1%) | 25.2 - 50.3 |  |
| Someone known for while | 58 (68.2%) | 57.2 - 77.9 | 27 (31.8%) | 22.1 - 42.8 |  |
| Recent acquaintance | 18 (78.3%) | 56.3 - 92.5 | 5 (21.7%) | 7.5 - 43.7 |  |
| ***Living separately from regular partner*(12/1,031)*** |  |  |  |  | *<0.001* |
| Same house | 51 (33.1%) | 25.8 - 41.1 | 103 (66.9%) | 58.9 - 74.2 |  |
| Same neighborhood | 188 (59.5%) | 83.9 - 65.0 | 128 (40.5%) | 35.0 - 46.1 |  |
| Same town/area | 213 (64.9%) | 59.5 - 70.1 | 115 (35.1%) | 29.9 - 40.5 |  |
| Same province | 92 (61.7%) | 53.4 - 69.6 | 57 (38.3%) | 30.4 - 46.6 |  |
| Different province | 46 (63.9%) | 51.7 - 74.9 | 26 (36.1%) | 25.1 - 48.3 |  |
| ***Transactional sex*(3/1,031) ¥*** |  |  |  |  | *0.160* |
| No | 547 (58.6%) | 55.3 - 61.7 | 387 (41.4%) | 38.3 - 44.7 |  |
| Yes | 41 (50.0%) | 38.7 - 61.3 | 41 (50.0%) | 38.7 - 61.3 |  |
| ***Intergenerational sex*(3/1,031) ¥*** |  |  |  |  | *0.016* |
| No | 373 (60.9%) | 57.0 - 64.8 | 239 (39.1%) | 35.2 - 43.0 |  |
| Yes | 222 (53.4%) | 48.4 - 58.2 | 194 (46.6%) | 41.8 - 51.6 |  |

***Missing data; ¥In the last 12 months**
