# Supplementary material for: Predictors of male condom use among sexually active heterosexual young women in South Africa, 2012
Source: BMC Public Health. 2018 Sep 24;18:1137. doi: 10.1186/s12889-018-6039-8 (PMC6154873; doi:10.1186/s12889-018-6039-8)
Supplement: Supplementary file 9 — Table S3. Access to media and exposure to HIV Communication Programmes among young women aged 16–24 years, National HIV Communication Survey, South Africa, 2012, Frequency distribution table showing Access to media and exposure to HIV Communication Programmes among Sexually Active Young Women in South Africa with percentages and Chi-square Inferences, Access to media and exposure to HIV Communication Programmes (DOCX 16 kb) [file 12889_2018_6039_MOESM9_ESM.docx]

| ***Variable*** | ***Condom use at last sex*** | | ***Non-condom use at last sex*** | | ***P-value (chi-square test)*** |
| --- | --- | --- | --- | --- | --- |
|  | *N (%)* | *95% CI* | *N (%)* | *95% CI* |  |
| **Overall condom use** | 595 (57.9%) | 54.8 - 60.2 | 433 (42.1%) | 39.1 - 45.2 |  |
| ***Radio*(3/1,031)*** |  |  |  |  | 0.103 |
| Never listened ¥ | 171 (54.1%) | 48.4 - 59.7 | 145 (45.9%) | 40.3 - 51.6 |  |
| Listened ¥ | 424 (59.6%) | 55.8 - 63.2 | 288 (40.4%) | 37.8 - 44.2 |  |
| ***TV*(3/1,031)*** |  |  |  |  | *0.042* |
| Never watched ¥ | 87 (50.9%) | 43.1 - 58.6 | 84 (49.1%) | 41.4 - 56.9 |  |
| Watched ¥ | 508 (59.3%) | 55.9 - 62.6 | 349 (40.7%) | 37.4 - 44.1 |  |
| ***Magazine*(3/1,031)*** |  |  |  |  | *0.684* |
| Never read ¥ | 347 (57.4%) | 53.3 - 61.3 | 258 (42.6%) | 38.7 - 46.7 |  |
| Read ¥ | 248 (58.6%) | 53.8 - 63.4 | 175 (41.4%) | 36.6 - 46.2 |  |
| ***Newspaper*(3/1,031)*** |  |  |  |  | *0.605* |
| Never read ***¥*** | 268 (58.8%) | 54.1 - 63.3 | 188 (41.2%) | 36.7 - 45.9 |  |
| Read ¥ | 327 (57.2%) | 53.0 - 61.3 | 245 (42.8%) | 38.7 - 47.0 |  |
| ***Internet*(3/1,031)*** |  |  |  |  | *<0.001* |
| Had no access ¥ | 362 (52.9%) | 49.1 - 56.7 | 322 (47.1%) | 43.3 - 50.9 |  |
| Had access ¥ | 233 (67.7%) | 62.5 - 72.6 | 111 (32.3%) | 27.4 - 37.5 |  |
| ***Intersexions*(3/1,031) ^(TV programme)^*** |  |  |  |  | *<0.001* |
| Not exposed ¥ | 227 (50.8%) | 46.0 - 55.5 | 220 (49.2%) | 44.5 - 54.0 |  |
| Exposed ¥ | 368 (63.3%) | 59.3 - 67.3 | 213 (36.7%) | 32.7 - 40.7 |  |
| ***4Play:Sex Tips for Girls*(3/1,031) ^(TV programme)^*** |  |  |  |  | *0.801* |
| Not exposed ¥ | 467 (58.1%) | 54.6 - 61.5 | 337 (41.9%) | 38.5 - 45.4 |  |
| Exposed ¥ | 128 (57.1%) | 50.4 - 63.7 | 96 (42.9%) | 36.3 - 49.6 |  |
| ***Brothers for Life*(3/1,031) ^(TV programme)^*** |  |  |  |  | *<0.001* |
| Not exposed ¥ | 238 (51.2%) | 46.5 - 55.8 | 227 (48.8%) | 44.2 - 53.5 |  |
| Exposed ¥ | 357 (63.4%) | 59.3 - 67.4 | 206 (36.6%) | 32.6 - 40.7 |  |
| ***Scrutinize*(3/1,031) ^(TV programme)^*** |  |  |  |  | *0.009* |
| Not exposed ¥ | 106 (50.0%) | 43.1 - 56.9 | 106 (50.0%) | 43.1 - 56.9 |  |
| Exposed ¥ | 489 (59.9%) | 56.4 - 63.3 | 327 (40.1%) | 36.7 - 43.5 |  |
| ***Soul City*(3/1,031) ^(TV programme )^*** |  |  |  |  | *0.199* |
| Not exposed ¥ | 240 (55.6%) | 50.7 - 60.3 | 192 (44.4%) | 39.7 - 49.3 |  |
| Exposed ¥ | 355 (59.6%) | 55.5 - 63.5 | 241 (40.4%) | 36.6 - 44.5 |  |
| ***loveLife*(3/1,031) ^(TV and radio programme)^*** |  |  |  |  | *0.471* |
| Not exposed ¥ | 560 (57.6%) | 54.4 - 60.7 | 412 (42.4%) | 39.3 - 45.6 |  |
| Exposed ***¥*** | 35 (62.5%) | 48.5 - 75.1 | 21 (37.5%) | 24.9 - 51.5 |  |
| ***Siyanqoba Beat it!*(3/1,031) ^(TV programme)^*** |  |  |  |  | *<0.001* |
| Not exposed ¥ | 247 (51.4%) | 46.8 - 55.9 | 234 (48.6%) | 44.1 - 53.2 |  |
| Exposed ¥ | 348 (63.6%) | 59.4 - 67.7 | 199 (36.4%) | 32.3 - 40.6 |  |
| ***We BEAT TB*(3/1,031) ^(TV programme)^*** |  |  |  |  | *0.001* |
| Not exposed ¥ | 188 (50.8%) | 45.6 - 56.0 | 182 (49.2%) | 44.0 - 54.4 |  |
| Exposed ¥ | 407 (61.9%) | 58.0 - 65.6 | 251 (38.1%) | 34.4 - 42.0 |  |
| ***i-Life*(3/1,031) ^(Radio programme)^*** |  |  |  |  | *0.025* |
| Not exposed ¥ | 571 (57.3%) | 54.1 - 60.4 | 426 (42.7%) | 39.6 - 45.9 |  |
| Exposed ¥ | 24 (77.4%) | 58.9 - 90.4 | 7 (22.6%) | 9.6 - 41.1 |  |

***Missing data; ¥In the last 12 months**
