# Supplementary material for: Predictors of male condom use among sexually active heterosexual young women in South Africa, 2012
Source: BMC Public Health. 2018 Sep 24;18:1137. doi: 10.1186/s12889-018-6039-8 (PMC6154873; doi:10.1186/s12889-018-6039-8)
Supplement: Supplementary file 10 — Table S4. Demographic and sexual behaviours determining HIV infection among sexually active young women aged 16–24 years, National HIV Communication Survey, South Africa, 2012, Factors associated with HIV Infection among Sexually Active Young Women in South Africa with percentages and Chi-square Inferences, Determinants of HIV Infection. (DOCX 15 kb) [file 12889_2018_6039_MOESM10_ESM.docx]

| **Demographic determinants of HIV** | | | |
| --- | --- | --- | --- |
|  | **HIV+ N (%)** | **HIV- N (%)** | **P-value** |
| **Overall self-reported HIV status** | **46 (6.3%)** | **681 (93.7%)** |  |
| ***Age group *(304/1,031)*** |  |  | ***0.057*** |
| 16 – 19 years | 5 (3.1%) | 156 (96.9%) |  |
| 20 – 24 years | 41 (7.2%) | 525 (92.8%) |  |
| ***Province *(304/1,031)*** |  |  | ***0.274*** |
| Eastern Cape | 2 (2.7%) | 73 (97.3%) |  |
| Free State | 8 (11.6%) | 61 (88.4%) |  |
| Gauteng | 7 (5.0%) | 132 (95.0%) |  |
| KwaZulu-Natal | 11 (7.3%) | 140 (92.7%) |  |
| Limpopo | 4 (7.4%) | 50 (92.6%) |  |
| Mpumalanga | 7 (10.6%) | 59 (89.4%) |  |
| North West | 1 (3.1%) | 31 (96.9%) |  |
| Northern Cape | 1 (10.0%) | 9 (90.0%) |  |
| Western Cape | 5 (3.8%) | 126 (96.2%) |  |
| ***Race *(304/1,031)*** |  |  | ***0.622*** |
| Black | 43 (6.8%) | 592 (93.2%) |  |
| Colored | 3 (3.4%) | 84 (96.6%) |  |
| Indian | 0 (0.0%) | 4 (100.0%) |  |
| White | 0 (0.0%) | 1 (100.0%) |  |
| ***Settlement type *(304/1,031)*** |  |  | ***0.33*** |
| Urban formal | 11 (4.6%) | 230 (95.4%) |  |
| Urban informal | 24 (8.0%) | 276 (92.0%) |  |
| Peri-urban | 7 (8.3%) | 77 (91.7%) |  |
| Tribal settlement | 3 (3.4%) | 86 (96.6%) |  |
| Farming | 1 (7.7%) | 12 (92.3%) |  |
| ***Housing type *(304/1,031)*** |  |  | ***0.216*** |
| Formal house | 7 (3.4%) | 197 (96.6%) |  |
| Mostly formal | 16 (8.3%) | 177 (91.7%) |  |
| Mostly informal | 16 (8.7%) | 169 (91.3%) |  |
| Squatter camp | 2 (2.8%) | 69 (97.2%) |  |
| Traditional house | 5 (7.9%) | 58 (92.1%) |  |
| Hostel | 0 (0.0%) | 9 (100.0%) |  |
| Other | 0 (0.0%) | 2 (100.0%) |  |
| ***Level of Education *(304/1,031)*** |  |  | ***0.003*** |
| Primary | 4 (26.7%) | 11 (73.3%) |  |
| Grade 11 | 27 (8.0%) | 312 (92.0%) |  |
| Matric | 14 (4.6%) | 289 (95.4%) |  |
| Tertiary | 1 (1.4%) | 68 (98.6%) |  |
| No school | 0 (0.0%) | 1 (100.0%) |  |
| ***Marital status *(309/1,031)*** |  |  | ***0.26*** |
| Single | 26 (7.7%) | 311 (92.3%) |  |
| Stable relationship | 11 (4.5%) | 232 (95.5%) |  |
| Cohabitation | 4 (4.4%) | 87 (95.6%) |  |
| Married | 5 (9.8%) | 46 (90.2%) |  |
| ***Employment status *(310/1,031)*** |  |  | ***0.298*** |
| Unemployed | 31 (7.0%) | 415 (93.0%) |  |
| Employed | 8 (8.1%) | 91 (91.9%) |  |
| Student | 7 (4.0%) | 169 (96.0%) |  |
| ***Socio-economic status *(304/1,031)*** |  |  | ***0.168*** |
| High | 7 (3.7%) | 181 (96.3%) |  |
| Medium | 25 (8.0%) | 289 (92.0%) |  |
| Low | 14 (6.2%) | 211 (93.8%) |  |
| ***Food security¥*** |  |  | ***<0.001*** |
| Insecure | 17 (14.2%) | 103 (85.8%) |  |
| Secure | 29 (4.8%) | 578 (95.2%) |  |
| **Sexual behaviours determining HIV** | | | |
| ***Multiple sexual partners *(304/1,031) ¥*** |  |  | ***<0.001*** |
| No | 34 (5.1%) | 629 (94.9%) |  |
| Yes | 12 (18.8%) | 52 (81.2%) |  |
| ***Sexual debut *(304/1,031)*** |  |  | ***<0.001*** |
| 0–15 years | 17 (15.3%) | 94 (84.7%) |  |
| 16–17 years | 19 (6.4%) | 279 (93.6%) |  |
| 18–19 years | 7 (2.9%) | 233 (97.1%) |  |
| 20–24 years | 3 (3.9%) | 75 (96.1%) |  |
| ***Transactional sex *(304/1,031) ¥*** |  |  | ***0.009*** |
| No | 38 (5.7%) | 634 (94.3%) |  |
| Yes | 8 (14.6%) | 47 (85.4%) | . |
| ***Intergenerational sex *(304/1,031) ¥*** |  |  | ***0.01*** |
| No | 19 (4.4%) | 413 (96.6%) |  |
| Yes | 27 (9.1%) | 268 (90.9%) |  |
